# Supplementary material for: A transphyletic study of metazoan β-catenin protein complexes
Source: Zoological Lett. 2024 Dec 2;10:20. doi: 10.1186/s40851-024-00243-y (PMC11613877; doi:10.1186/s40851-024-00243-y)
Supplement: Supplementary file 15 — Supplementary Material 15 [file 40851_2024_243_MOESM15_ESM.docx]

Table S4

| **Plasmid** | **Description** | **Source** |
| --- | --- | --- |
| pCS2+ Flag-Nvec-β-cat | Flag-tagged *N. vectensis* β-catenin | This study |
| pCS2+ Flag-Xlae-β-cat | Flag-tagged *X. laevis* β-catenin | This study |
| pCS2+ Flag-Eflu-β-cat | Flag-tagged *E. fluviatilis* β-catenin | This study |
| pCS2+ Flag-Bmik-β-cat | Flag-tagged *B. mikado* β-catenin | This study |
| pCSf107 +β-gal | β- galactosidase | Yuuri Yasuoka |
| 8x Super TOPFLASH (Addgene #12456) | Beta-catenin reporter. TCF/LEF sites upstream of a luciferase reporter. | Veeman et al. (2003) |
| 8x Super FOPFLASH (Addgene #12457) | Contains mutated TCF binding sites upstream of a luciferase reporter. | Veeman et al. (2003) |

**References:**

Veeman et al. (2003). Zebrafish prickle, a modulator of noncanonical Wnt/Fz signaling, regulates gastrulation movements. Current Biology, 13(8), 680–685.
